# Effect of reduced pH on physiology and shell integrity of juvenile *Haliotis iris* (pāua) from New Zealand

**DOI:** 10.7717/peerj.7670

**Published:** 2019-09-25

**Authors:** Vonda J. Cummings, Abigail M. Smith, Peter M. Marriott, Bryce A. Peebles, N. Jane Halliday

**Affiliations:** 1 National Institute of Water and Atmospheric Research, Wellington, New Zealand; 2 Department of Marine Science, University of Otago, Dunedin, New Zealand

**Keywords:** Ocean acidification, pH, Mollusc, Juvenile, Coastal marine, CO_2_, Shell thickness, Warming

## Abstract

The New Zealand pāua or black footed abalone, *Haliotis iris*, is one of many mollusc species at potential risk from ocean acidification and warming. To investigate possible impacts, juvenile pāua (~24 mm shell length) were grown for 4 months in seawater pH/pCO_2_ conditions projected for 2100. End of century seawater projections (pH_T_ 7.66/pCO_2_ ~1,000 μatm) were contrasted with local ambient conditions (pH_T_ 8.00/pCO_2_ ~400 μatm) at two typical temperatures (13 and 15 °C). We used a combination of methods (morphometric, scanning electron microscopy, X-ray diffraction) to investigate effects on juvenile survival and growth, as well as shell mineralogy and integrity. Lowered pH did not affect survival, growth rate or condition, but animals grew significantly faster at the higher temperature. Juvenile pāua were able to biomineralise their inner nacreous aragonite layer and their outer prismatic calcite layer under end-of-century pH conditions, at both temperatures, and carbonate composition was not affected. There was some thickening of the nacre layer in the newly deposited shell with reduced pH and also at the higher temperature. Most obvious was post-depositional alteration of the shell under lowered pH: the prismatic calcite layer was thinner, and there was greater etching of the external shell surface; this dissolution was greater at the higher temperature. These results demonstrate the importance of even a small (2 °C) difference in temperature on growth and shell characteristics, and on modifying the effects at lowered pH. Projected CO_2_-related changes may affect shell quality of this iconic New Zealand mollusc through etching (dissolution) and thinning, with potential implications for resilience to physical stresses such as predation and wave action.

## Introduction

Increased greenhouse gas emissions since the industrial revolution have already resulted in significant warming and acidification of oceanic waters (Caldeira & Wickett, 2003; Orr et al., 2005). Ocean acidification, the term used to describe the change in ocean chemistry caused by excess atmospheric CO_2_ dissolving in seawater, reduces seawater pH and carbonate ion concentrations. Both changes are a concern for marine organisms as disruption of their acid-base balance may alter physiological functions, and those with calcium carbonate shells and/or skeletons may experience dissolution of these structures (Raven et al., 2005; Doney et al., 2009; Ries, Cohen & McCorkle, 2009). Understanding the implications of ocean acidification and warming to ecologically important species, ecosystem diversity and ecosystem function is a current challenge (Widdicombe & Spicer, 2008; Parker et al., 2013). Molluscs have been well-studied in this context (Gazeau et al., 2007, 2013; Miller et al., 2009; Parker et al., 2013) in part because they constitute an important component of the global food economy that will be affected by these changes (FAO, 2009, 2018). Shellfish fisheries have already been demonstrably affected by ocean acidification (Barton et al., 2012, 2015).

The majority of marine molluscs are found in coastal areas (Gazeau et al., 2013) which experience considerable natural variation in temperature and pH (Hofmann et al., 2011; Law et al., 2018). Over the next decades global (ocean acidification and warming) and local (e.g. freshwater runoff) environmental perturbations are expected to affect both the extremes and the normal ranges of pH and temperature in coastal marine systems (Kapsenberg & Hofmann, 2016). Reduced seawater pH and carbonate concentrations may result in a loss of fitness (Thomsen & Melzner, 2010; Stumpp et al., 2011) as energy is diverted away from growth and reproduction to ensure that basic metabolic processes are maintained (Clarke, 1987; Pörtner, 2008). This may in turn affect the organisms ability to cope with other environmental stressors (Gazeau et al., 2007; Cummings et al., 2011) and, ultimately, influence its survival (Pörtner, 2008; Guy et al., 2014). Effects on behaviour have also been noted, including those that increase susceptibility to predation (Watson et al., 2014; Wright et al., 2018; Kong et al., 2019).

Ocean acidification studies to date have revealed complex and variable biological responses across a wide range of taxa (Fabry et al., 2008; Kroeker et al., 2010, 2013). Variable responses have been noted even within closely related species (sea urchins, Dupont & Thorndyke, 2009; *Crassostrea* spp., Miller et al., 2009; *Haliotis* spp., Byrne et al., 2010, 2011; Crim, Sunday & Harley, 2011; Kimura et al., 2011), or for the same species originating from different regions (Waldbusser, Bergschneider & Green, 2010). Thus, while molluscs are recognised as one of the groups that are most susceptible to ocean acidification (Gazeau et al., 2013; Kroeker et al., 2013), it is at the species or even the population-level, where the complex systems of resilience and response to environmental conditions are enacted (Pespeni et al., 2013). While effects on all life stages have been investigated, more emphasis has been placed on early development due to their recognised vulnerability to environmental perturbations (Raven et al., 2005; Byrne et al., 2011; Parker et al., 2013). Results have shown larval and juvenile stages are indeed generally more negatively affected than later stages (Waldbusser, Bergschneider & Green, 2010; Gazeau et al., 2013; Kroeker et al., 2013; Parker et al., 2013). Nevertheless, negative effects on calcification, growth and metabolism of adults have also been demonstrated (Cummings et al., 2011; Scanes et al., 2014).

Molluscs are vigorous and controlling calcifiers (Lowenstam & Weiner, 1989) that can generate their shell via internal metabolic processes rather than directly using the surrounding seawater (Wicks & Roberts, 2012). Nevertheless, the energetic cost to the organism in maintaining pH of the fluid at the site of calcification under lowered pH conditions (Gazeau et al., 2013) will likely also affect essential functions (Thomsen & Melzner, 2010; Navarro et al., 2013; Waldbusser et al., 2016). Additionally, the particular calcium carbonate polymorph of the shell influences its susceptibility to dissolution under reduced pH conditions (Ries, Cohen & McCorkle, 2009), with calcite considered the most robust as carbonate ion concentrations decline. Most molluscs shells are protected from direct contact with the surrounding seawater by a thin organic periostracum that covers the external shell surface (Saleuddin & Petit, 1983; Ries, Cohen & McCorkle, 2009; Thomsen et al., 2010). However, mechanical damage or disruption to this periostracum does occur (Hurd et al., 2011), rendering the underlying shell more susceptible to dissolution.

Like many molluscs, gastropods in the family Haliotidae are important in maintaining healthy ecosystem function and influencing seafloor diversity. Globally, there are several studies on abalone (*Haliotis* spp.) responses to ocean acidification, mostly focused on larvae (Byrne et al., 2010; Crim, Sunday & Harley, 2011; Kimura et al., 2011) and young juveniles (Cunningham, Smith & Lamare, 2016; Kim, Barry & Micheli, 2013; Li et al., 2018). For larvae reared under reduced pH conditions, rates of abnormal development were higher, sizes of normally developed larvae were smaller, and calcification and thermal tolerances were reduced (Zippay & Hofmann, 2010; Byrne et al., 2011; Kimura et al., 2011). Survival and growth of juveniles ranging in size from newly settled to 40 mm shell length (SL) were negatively affected, with shell erosion, decreased shell weight (SW), and altered biochemical composition also noted (Kim, Barry & Micheli, 2013; Cunningham, Smith & Lamare, 2016; Li et al., 2018). Few of these studies have considered effects on the shell in detail, however.

The pāua or black-footed abalone *Haliotis iris* Gmelin, 1791 is widespread in New Zealand coastal ecosystems, and of considerable ecological, economic and cultural value. Endemic to New Zealand, it is found subtidally to intertidally on coasts around the North Island, the South Island, Stewart Island and the offshore Chatham Islands. Pāua have been an important taonga (treasure) and food source for Māori for 800 years (Smith, 2011), are considered a local delicacy, and are wild-harvested and farmed for domestic and export markets (export meat value alone is ~$36M; New Zealand Seafood Exports, 2017). Additionally, their colourful and patterned shell is an iconic resource that is used widely for jewellery and art. To investigate their potential response to projected ocean acidification, juvenile pāua were exposed to ambient and lowered pH seawater for 4 months. We assessed whether lowered seawater pH alters physiological characteristics (survival, growth, condition) and/or shell integrity (thickness, density, composition). Given the projections for ocean warming and the important influence of temperature on growth, we also investigated how a small variation in temperature would modify the effect of lower pH.

## Materials and Methods

*Haliotis iris* were grown for 4 months (121 days) in two different pH conditions (Table 1) and their responses evaluated at the end of this period. Throughout this manuscript, pH is presented on the total hydrogen ion scale (pH_T_) at in situ temperature. Our chosen pH_T_ levels were ~8.00 (ambient Wellington Harbour pH), and 7.66 (within the projected of 0.3–0.5 pH units decline in the open ocean by 2100; Caldeira & Wickett, 2003; Orr et al., 2005; Doney, 2010; and 0.335 for the New Zealand region, by Law et al., 2018). A recent 3-year coastal observation record reveals that pH_T_ in Wellington Harbour naturally ranges from 7.9 to 8.15 annually. Temperatures at this location ranged from 8 to 20 °C over the same time period (pH and temperature data: New Zealand Ocean Acidification Observing Network (NZOA-ON), 2015). Two seawater temperatures were used in our experiment (13 and 15 °C), that are within the range of temperatures naturally experienced in Wellington Harbour in autumn and spring, respectively. The differences between these temperatures also represent the projected magnitude of change in average sea surface temperatures for end-century in southern New Zealand locations (+2.5 °C under Representative Concentration Pathway 8.5; Law et al., 2018).

**Table 1:** Seawater conditions for each experimental treatment. Calcite and aragonite saturation states (Ω_Ca_ and Ω_Ar_, respectively) and pCO_2_ are calculated values from the measured pH_T_, and the alkalinity (A_T_ ) measured in February 2011 (A_T_ = 2,228.3 ± 1.6; *N* = 11) at a salinity of 34.5. Data presented for pH_T_ are averages ± SE.

Temperature (°C)	pH_T_*	Ω_Ca_	Ω_Ar_	pCO_2_
12.60 ± 0.01	8.03 ± 0.00	3.27	2.09	395
	7.66 ± 0.00	1.52	0.97	1,023
14.89 ± 0.00	8.00 ± 0.00	3.34	2.14	432
	7.66 ± 0.00	1.66	1.07	1,041

**Note:**

* *N* > 730 spectrophotometer-based pH measurements made for each treatment header tank over the 4 months experiment (~4 hourly sampling).

### Experimental set up

#### Haliotis iris juveniles

Juvenile *H. iris* used in the experiment were hatchery-sourced and were 14 months old at the beginning of the experiment. *H. iris* were kept in experimental chambers (36 mm wide × 36 mm deep × 285 mm long; six replicates per treatment), through which seawater flowed at a constant rate of 140 ml min^−1^, ensuring complete turnover of seawater approximately every 3 min (thus ensuring the pāua would not modify the seawater pH via respiration). A total of 10 live individuals were placed in each of the six replicate chambers, with replicates from the four treatments arranged in a randomised block design. A maximum of two randomly selected individuals from each chamber were used for analysis (as detailed below). To avoid shocking the juveniles by immediately placing them in the experimental pH and temperature conditions, temperature was first changed slowly over 3 weeks, the animals left at these temperatures for 2 weeks, and the pH then gradually altered to target levels over a further 1 week period. Pāua were fed at regular intervals throughout the experiment, ad libitum. During the acclimation period, *H. iris* were fed freshly collected red seaweed (*Porphyra* sp.). Once the experimental conditions were reached, *H. iris* were fed commercially produced pellets (ABMAX; E.N. Hutchinson Ltd, Auckland, New Zealand). This diet shift resulted in a change in shell colouration and enabled us to clearly distinguish shell grown over the 4 month experiment. Both feed types are routinely used in New Zealand abalone hatcheries, and pāua grow well under both regimes.

#### Seawater manipulation and measurement

Seawater pH was manipulated via CO_2_ diffusion from silicon coils submerged in 60 l header tanks (controlled by Omega PHCN-37 pH controllers connected to Hamilton Liq-Glass pH probes). Probes were calibrated regularly with TRIS and AMP buffers, and measures were validated multiple times per day in water samples using an automated spectrophotometric system and thymol blue dye (McGraw et al., 2010). Temperature was altered using heater elements (controlled by Omega CN-740 temperature controllers connected to precision, pre-calibrated PT100 probes). One header tank was manipulated for each treatment, due to logistical reasons; however, none of our measurements or observations suggested that there was anything substantially different about the header tanks (indicating, for example, a contamination problem) other than the CO_2_ and temperature dose treatments we applied. Additionally, the four header tanks were unable to influence each other, and were identical in that they were continually supplied by seawater and CO_2_ from single common sources (Hurlbert, 2013). Thus, in our analyses we consider the replicate chambers assigned to a given treatment type as independent- rather than pseudo-replicates (Hurlbert, 1984; Cornwall & Hurd, 2016).

Aragonite and calcite saturation states (Ω_Ar_ and Ω_Ca_, respectively) and the partial pressure of CO_2_ in equilibrium with the sample (pCO_2_) were calculated from measured alkalinity (A_T_) and pH_T_, at the average experimental water temperature and salinity, using the refitted Mehrbach et al. (1973) equilibrium constants (Dickson & Millero, 1987). A_T_ was measured using a closed cell potentiometric titration method (Dickson, Sabine & Christian, 2007) from water samples (preserved with HgCl_2_) taken from each treatment header tank. The accuracy of this method is estimated to be 1.5 μmol kg^−1^, based on the analyses of Certified Reference Material.

### *Haliotis iris* physiological response

We assessed survival at weekly intervals, and growth and physiological condition at the end of the experiment. Survival assessments included all individuals in each replicate, while all other responses were made on one or two randomly selected individuals per replicate. The absolute growth of live pāua, identifiable from the blue coloured shell, was measured to the nearest mm (from photographs with a scale-bar included, using Image-J) and expressed as a daily growth rate (μm d^−1^). The relative growth rate (% change in SL relative to the initial SL) was also calculated, using the formula of Hopkins (1992). Physiological condition was calculated using the ratio of dry flesh weight (FW; dried at 60 °C for 48 h) to dry SW (air dried for 48 h), following Lucas & Beninger (1985; FW/SW*100). The resultant condition index (CI_FW:SW_) provides an integration of the present metabolic state of the organism (Mann, 1978; Roper et al., 1991). Simple allometric relationships between SW and SL were also examined. One individual per replicate was available for the evaluations involving FW, and two individuals per replicate for those involving SW.

### Shell morphology and integrity

**Figure 1:**
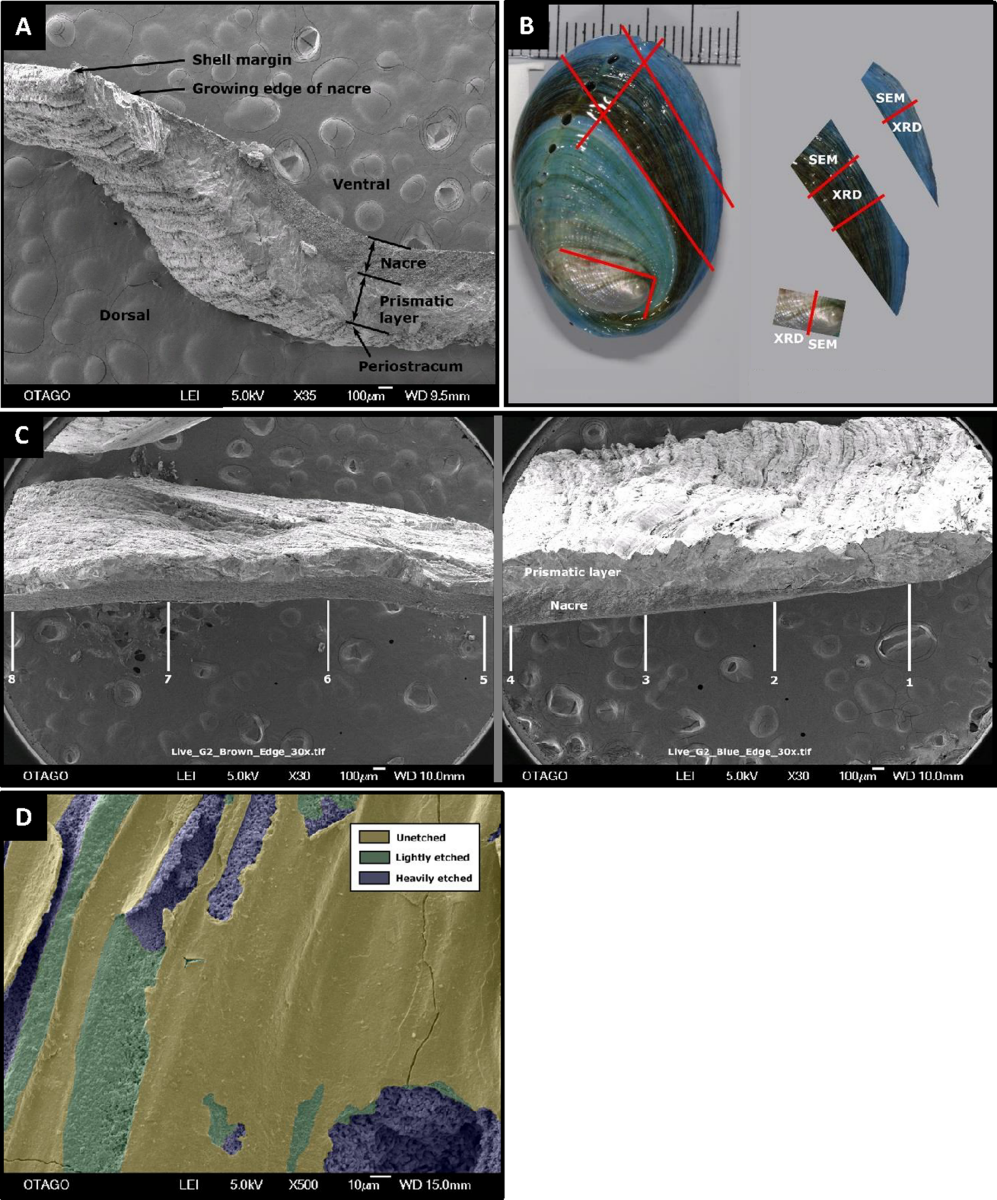
Pāua shell structure and analytical protocols. General pāua shell structure (A), and the various protocols used for analysis in this study (B and C). (A) Cross-section of pāua shell showing layers. (B) Schematic showing the sectioning procedure used for the juvenile pāua shells (indicated by red lines). The experiment (blue) shell was laid down during the experiment. The pre-experiment (brown) shell was deposited during the acclimation phase, prior to the experiment start. The spire (silver) shell is earliest formed portion of the shell. SEM, portion of shell used for SEM and SEM-EDS analyses; XRD, portion of shell used for X-ray diffraction analysis. (C) The placement of the eight sampling sites across cross sections of the pre-experiment shell (positions 5–8) and the experiment shell (positions 1–4), at which the prismatic and nacre layer thicknesses were quantified using SEM. (D) Artificially coloured SEM image depicting areas of unetched, lightly etched and heavily etched dorsal shell surfaces.

To evaluate changes in morphology and integrity of the pāua shells over the course of the experiment, shells from one pāua from each of the six treatment replicates were examined (with the exception of the pH 8.00/15 °C treatment: only five replicates were analysed). Whole shells were first cleaned in MilliQ water in an ultrasonic bath, to remove detritus from the shell surfaces, and to partially hydrate the organic matrix in the shell (Sun & Bhushan, 2012) making it less prone to shattering during sectioning. Precision cuts were then made on the surface (Fig. 1B) that did not fully penetrate the shell, in order to isolate the three specific portions for subsequent sampling: new shell grown during the experiment (blue), pre-experiment shell (brown), and the spire (oldest part of the shell). Once dry the shells were fractured by bending, using forceps with thick soft material on the inner faces to prevent physical damage to the surfaces. The different shell sections were analysed using X-ray diffractometry (XRD), scanning electron micrographs (SEM) and energy-dispersive X-ray spectroscopy (EDS), as described below.

**Figure 2:**
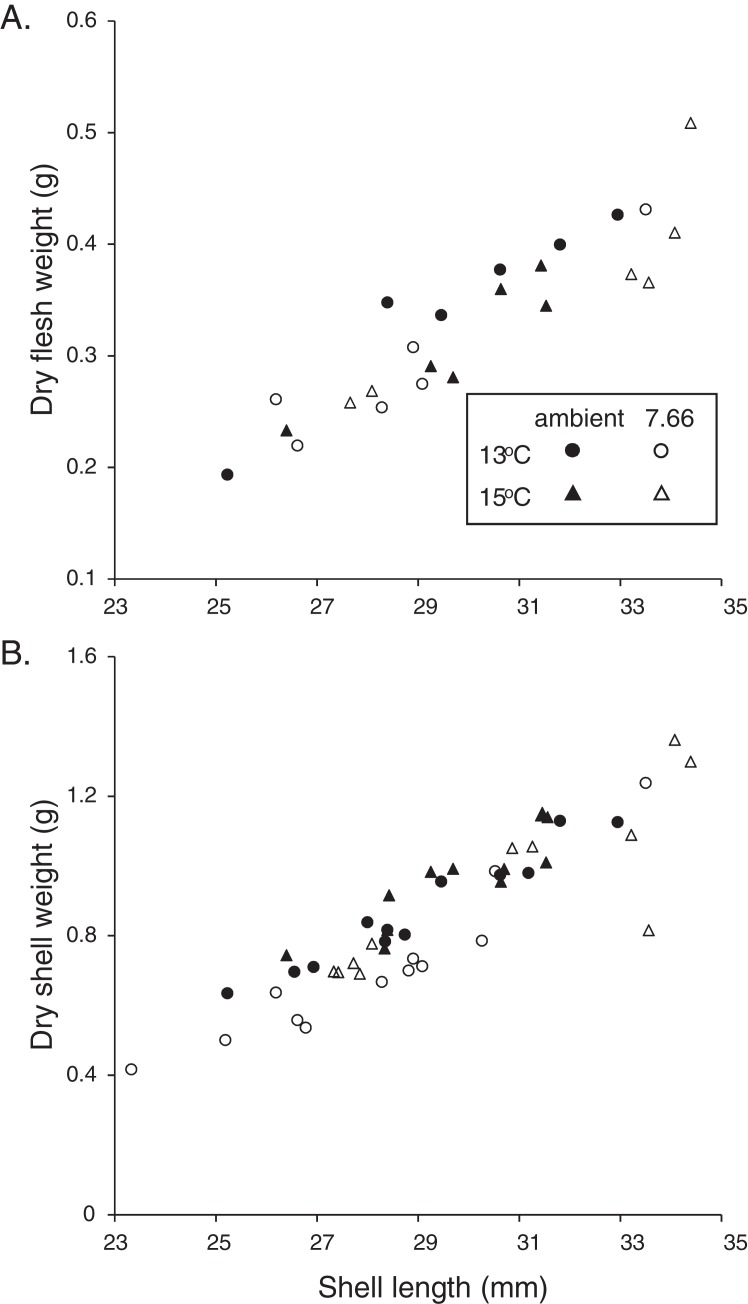
Allometric relationships in pāua from each experimental treatment. Relationship of pāua shell length with (A) dry flesh weight (*N* = 6) and (B) dry shell dry weight (*N* = 12).

Shell ultrastructure, thickness and surface characteristics were assessed using SEM. Each shell section was coated with gold-palladium and photographed in a JEOL SEM-2200FS Cryo-TEM on the cross section, dorsal and ventral surfaces at multiple scales. The SEM cross section for the experimental (new) growth portion always included the growing margin of the shell, to ensure consistent representative measurements of layer thicknesses along the shell. Cross-sections at 30× magnification were used to assess total shell thickness, as well as thicknesses of the (inner) nacre and (outer) prismatic layers (Figs. 2A and 2C). For each shell, layer thickness was assessed at eight positions across the shell—four across the experiment phase growth (labelled 1–4) and four positions in the pre-experiment acclimation phase (labelled 5–8), using ImageJ/Fiji (Schindelin et al., 2012) (Fig. 1C). Imaged positions were approximately 1.0–1.3 mm apart, starting at the growing edge (i.e. position 1). At the growing edge, the nacre had not yet formed; hence the nacre thickness is zero μm.

The degree of etching of the outer (dorsal) shell surface was assessed on 500× magnification images of the newly deposited and pre-experiment shells, each incorporating an area of 4,610 μm^2^ of shell surface. The proportion of the shell that was unetched, lightly etched or heavily etched was quantified using ImageJ (Fig. 1D), as a percentage of the total imaged area. Finally, crystal ultrastructure, size and formation were examined using SEM micrographs of cross sections and surfaces taken at 3,000× magnification.

### Shell composition (mineralogy)

Changes in mineral composition of shells were examined (one individual per replicate). Bulk skeletal composition (calcite:aragonite ratio, and wt% MgCO_3_ in calcite) was determined using XRD, while a higher spatial resolution EDS analysis was used to determine quantitative elemental composition (wt% calcium and wt% magnesium in the carbonate). For XRD analysis samples were powdered to crystallites, spiked with 0.1 g halite (NaCl) as an internal standard, and smeared onto a glass slide (per Gray & Smith, 2004). The position of the calcite peak (near 29.4 °2θ) was corrected using the known position of the halite peak at 31.72 °2θ, and wt% MgCO_3_ in calcite was calculated using the machine-specific calibration equation of Gray & Smith (2004), after the methods of Chave (1952, 1954). Wt% calcite in carbonate was calculated using the Peak Height Ratio method (Gray & Smith, 2004). For EDS analysis, shell fragments were coated in Au/Pd and a four mm^2^ area of the shell surface examined. The same locations on each specimen were selected: (a) based on the number of counts/second recorded (>1,500 counts/s is required to provide an accurate sample), and (b) to avoid clustering of readings on the surface. Each scan ran for 100 s. Percentages of the target elements calcium and magnesium present in the sampling area were generated from comparisons to factory standards.

### Statistical analyses

Differences in each response variable between treatments were assessed using two-way crossed ANOVA after assumptions were satisfied, with pH, temperature and a pH*temperature interaction term included. Assumptions were checked by examining the residual distribution plots and residuals vs predicted values and quantiles and using the Shapiro–Wilk test for normality. Any variables for which these suggested non-normality or heterogeneity of variance were rank transformed prior to analysis. Actual differences among treatment means were determined using Duncan’s multiple comparisons tests, where no significant interaction was detected. If a significant interaction term was found, multiple comparisons determined the effect of pH at each temperature separately, and temperature at each pH separately. All analyses were conducted using SAS software (SAS Institute), with *p* < 0.05 used to indicate a statistically significant response.

## Results

Distinct treatments of pH and temperature were successfully maintained at target levels for the 4 months of the experiment (Table 1). At the beginning of the experiment, the SL of the individual pāua averaged 23.5 ± 0.3 mm, and were similar across the different treatments (i.e. 13 °C/pH_T_ 8.00: 23.8 ± 0.6 mm; 13 °C/pH_T_ 7.66: 23.6 ± 0.7 mm; 15 °C/pH_T_ 8.00: 22.9 ± 0.6 mm; 15 °C/pH_T_ 7.66: 23.7 ± 0.9 mm), with no statistically significant differences detected (pH *p* = 0.6425, temp *p* = 0.5474, pH*temp *p* = 0.4479).

### *Haliotis iris* physiological response

The majority of the pāua survived the experiment, with only five mortalities across all treatments. These included three individuals from the lowered pH, 15 °C treatment and one each from the ambient pH treatments at each temperature. All the pāua grew measurably in the experimental conditions (Table 2). The average amount of new growth ranged from 5,273.5 ± 860.8 μm in the lowered pH, 13 °C treatment (a 21% increase in SL over the experiment; 44 μm d^−1^), to 8,837.5 ± 578.5 μm in the ambient pH, 15 °C treatment (a 34% increase; 73 μm d^−1^). Although growth was lower at pH_T_ 7.66 than at ambient pH, at each temperature (Table 2), this was not statistically significant. Both the amount of new growth and the percentage growth were significantly greater at 15 °C than at 13 °C (Table 2; new growth: pH *p* = 0.1844, temp *p* = 0.0003, pH*temp *p* = 0.4486; % growth: pH *p* = 0.3290, temp *p* = 0.0002, pH*temp *p* = 0.5337). Physiological condition (CI_FWSW_) was reduced at 15 °C, but was not affected by pH (Table 2; pH *p* = 0.0684, temp *p* = 0.0367, pH*temp *p* = 0.5733). Dry FW (normalised by SL) of the pāua from the ambient pH/13 °C treatment was slightly higher than those in the other treatments, although not significantly so (Fig. 2A; CI_FWSL_ pH *p* = 0.6272, temp *p* = 0.8583, pH*temp *p* = 0.1202).

**Table 2:** Growth and condition of pāua in the different pH and temperature treatments. The absolute growth (width of the new shell) and the percentage growth, and the condition (CI) of the live individuals over the 4 months experiment. Values presented are averages ± standard errors of six replicates per treatment. SL, shell length; CI_FW:SW_, flesh weight:shell weight; CI_SW:SL_, shell weight:shell length; CI_FW:SL_, flesh weight:shell length.

Temp (°C)	pH	New growth (μm)	% Growth (SL increase)	Growth rate (μm d^−1^)	CI_FW:SW_ (%)	CI_SW:SL_ (%)	CI_FW:SL_ (%)
13	Ambient	5,647.5 ± 450.0	21.8 ± 2.3	46.7	36.7 ± 1.7	3.0 ± 0.1	1.2 ± 0.1
	7.66	5,273.5 ± 860.8	20.9 ± 3.5	43.6	39.0 ± 1.0	2.5 ± 0.2	1.0 ± 0.1
15	Ambient	8,837.5 ± 578.5	34.3 ± 3.2	73.0	32.4 ± 1.4	3.2 ± 0.1	1.1 ± 0.1
	7.66	7,503.0 ± 515.8	27.9 ± 1.3	62.0	36.3 ± 2.1	3.0 ± 0.2	1.1 ± 0.1

### Shell morphology and integrity

The relationship between SW and SL in the juvenile *H. iris* is shown in Fig. 2B. Individuals from the lowered pH treatments had lighter shells for a given SL, a pattern that was true at both 15 and 13 °C (Fig. 2B). CI_SW:SL_ was significantly lower at pH_T_ 7.66 than ambient pH_T_ 8.00, and also at 13 °C than 15 °C (pH *p* = 0.0129, temp *p* = 0.0127, pH*temp *p* = 0.3623; Table 2).

**Figure 3:**
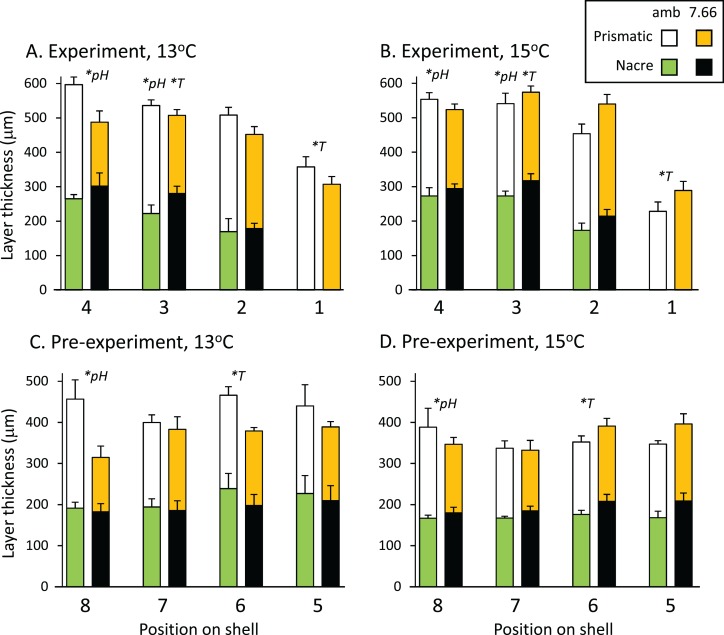
Pāua shell layer thicknesses from each pH and temperature treatment. Stack plots showing the thickness of the prismatic (top bars) and nacre (bottom bars) shell layers assessed at four positions across (A) the experimental shell at 13 °C and (B) 15 °C, and (C) the pre-experiment shell at 13 °C and (D) 15 °C. Statistically significant effects of treatment at a position are indicated by *pH or *T (temperature); please see text for more details.

The total thickness of the shells, measured from the SEM images, ranged from 300 to 600 μm on average, and did not differ significantly between treatments at any of the eight positions assessed (*p* > 0.05 in all cases). Figures 3A and 3B shows the prismatic and nacre layer thickness across the experimental shell. Comparisons revealed variable effects of pH and/or temperature at positions 4, 3 and 1. At position 4 the prismatic layer was significantly thinner at pH_T_ 7.66 than at ambient pH_T_ 8.00 (pH *p* = 0.0004, temp *p* = 0.8796, pH*temp *p* = 0.0567). At position 3, individuals from lower pH also had a thinner prismatic layer (pH *p* = 0.0315, temp *p* = 0.7088, pH*temp, *p* = 0.0883), as well as a thicker nacre layer (pH *p* = 0.0203, temp *p* = 0.0431, pH*temp *p* = 0.7338). The nacre at position 3 was also thinner at 13 °C than at 15 °C. At the growing edge (position 1) where the nacre layer had not yet formed, the prismatic layer was thicker at 13 °C than at 15 °C but was not affected by pH (pH *p* = 0.8537, temp *p* = 0.0124 pH*temp *p* = 0.0506; Figs. 3A and 3B). In the pre-experiment shell at position 8 (Figs. 3C and 3D) the prismatic layer was thicker at ambient pH than at pH_T_ 7.66 (pH *p* = 0.0192, temp *p* = 0.9019; pH*temp = 0.2956). At position 6, at ambient pH only, the nacre layer was thicker at 13 °C (pH*temp *p* = 0.0326; temp at pH_T_ 8.00 *p* = 0.0324, temp at pH_T_ 7.66 *p* = 0.3473; pH at 13 °C *p* = 0.1548, pH at 15 °C *p* = 0.1187).

**Figure 4:**
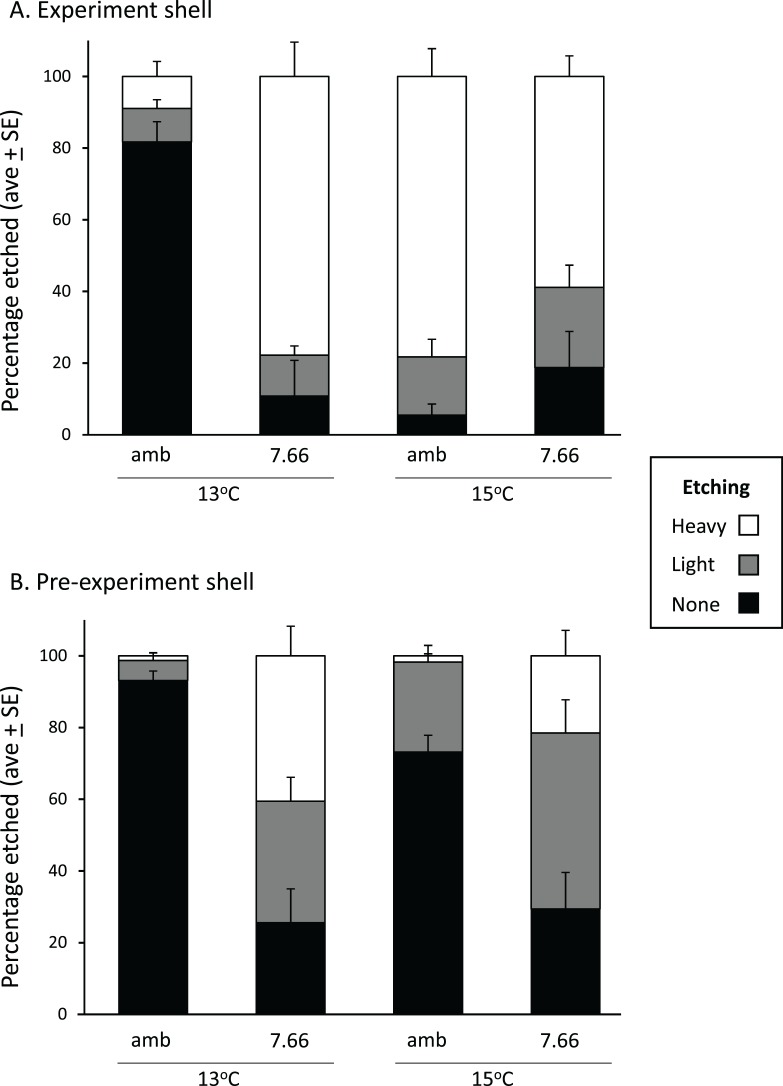
Pāua shell surface etching from each experimental treatment. Stack plots showing the degree of etching on the dorsal surface of (A) the experimental shell and (B) the pre-experiment shell, at each pH and temperature, determined from the analysis of ×500 magnification SEM images.

In the experiment shell, the amount of heavy etching on the dorsal surface was not consistent across temperatures or pH levels, respectively (pH*temp, *p* < 0.0001; Fig. 4A). Considerably more heavy etching was noted at pH_T_ 7.66 than pH_T_ 8.00 at 13 °C (by around 70%; *p* = 0.0002), but the opposite effect was found at 15 °C (by around 20%; pH_T_ 8.00 > pH_T_ 7.66; *p* = 0.0291). At ambient pH only, the percentage of heavily etched shell was greater at 15 °C than 13 °C (by around 70%; *p* = 0.0001) while at lowered pH the opposite effect was noted (13 > 15 °C *p* = 0.0254) (Fig. 4A). All experimental shells were lightly etched regardless of treatment (Fig. 4A; pH *p* = 0.3542, temp *p* = 0.0533, pH*temp *p* = 0.6517). In the pre-experiment shell, the percentage of heavily etched shell was significantly greater at the lower pH regardless of temperature (Fig. 4B; pH *p* < 0.001, temp *p* = 0.1056, pH*temp *p* = 0.0903). The percentage of lightly etched shell was influenced by both pH and temperature, being considerably greater at pH_T_ 7.66 and also at 15 °C (Fig. 4B; pH *p* = 0.0005; temp *p* = 0.0117, pH*temp *p* = 0.7358).

Our examination of the many SEM cross sectional and surface images did not reveal any differences in, or disruption to the ultrastructure of the shell.

### Shell composition (mineralogy)

The percent of calcite (and thus aragonite, as pāua are bimineralic) in the shell, determined from XRD, differed with the age of the shell. The spire (oldest shell) had the least calcite (8–23%; Table 3), whereas the experiment (new) shell and the pre-experiment shell contained appreciable amounts of very low-magnesium calcite (68–78% and 58–65%, respectively). In the experiment shell the wt% calcite was 5–6% higher at ambient pH_T_ 8.00 than at pH_T_ 7.66, and was not affected by temperature (Table 3; pH *p* = 0.0313, temp *p* = 0.0628, pH*temp *p* = 0.7464). There was no effect of treatment on the wt% calcite in either the pre-experiment shell (pH *p* = 0.6355, temp *p* = 0.2661, pH*temp *p* = 0.6355) or in the spire shell (pH *p* = 0.2604, temp *p* = 0.1674, pH*temp *p* = 0.5294).

**Table 3:** Calcite content (weight %) in the different areas of the pāua shell. Weight % calcite was determined using XRD. Experiment shell was generated during the exposure to experimental treatments, pre-experiment shell was generated in the acclimation phase, and the spire shell is the oldest part of the shell (the ‘whorl’). There was significantly more calcite in the ambient pH treatments of the experiment shell (*p* = 0.0313).

Temp (°C)	pH	Experiment shell	Pre-experiment shell	Spire shell
13	Ambient	77.99 ± 2.47	65.00 ± 2.24	9.73 ± 5.29
	7.66	71.72 ± 2.92	63.19 ± 6.16	22.62 ± 5.37
15	Ambient	72.54 ± 1.87	60.51 ± 1.88	8.13 ± 6.51
	7.66	67.83 ± 2.08	58.44 ± 4.32	10.30 ± 6.10

The effect on wt% calcium in skeletal carbonate of the experiment shell, determined using SEM-EDS, was not consistent across treatments (pH*temp = 0.0010), with an effect of temperature observed only at ambient pH (15 > 13 °C; pH_T_ 8.00 temp *p* = 0.0002; pH_T_ 7.66 temp *p* = 0.4081), and an effect of pH only at 13 °C (pH_T_ 7.66 > 8.00; temp 13 °C pH *p* = 0.0029; temp 15 °C pH = 0.0519). The same was noted for the pre-experiment shell (pH*temp *p* = 0.0277; pH_T_ 8.00 temp *p* = 0.0422; pH_T_ 7.66 temp *p* = 0.2017; temp 13 °C pH *p* = 0.0001; temp 15 °C pH = 0.1931). Wt % calcium in the spire shell was significantly greater at the lowered pH and the higher temperature (pH_T_ 7.66 > 8.00 *p* = 0.0466, temp 15 > 13 °C *p* = 0.0055, pH*temp = 0.5779).

X‐ray diffractometry results showed that the magnesium content in our juvenile pāua shell calcite is very low: always less than 2 wt% MgCO_3_ and mostly less than 1%. EDS samples, too, showed very low magnesium content. In both cases, the values were close to the detection limit for this method (0.5 in XRD, 0.25 in EDS); consequently, we did not conduct any statistical analyses on these data.

## Discussion

This study provides new information on how lowered seawater pH influences shell characteristics and physiology of juvenile pāua. Responses of juveniles after 4 months exposure to seawater pH levels projected for the end-of-century (pH_T_ 7.66, pCO_2_ ~1,000 μatm) were contrasted with those in present-day ambient conditions (pH_T_ ~8.00, pCO_2_ ~400 μatm). By conducting the investigations at 13 and 15 °C (present day averages for autumn and spring, respectively), the influence of a small variation in temperature on the responses to reduced pH was also able to be investigated.

### *Haliotis iris* physiological response

While we may have anticipated that exposure to lower pH would result in higher mortality, slower growth and reduced physiological condition in juvenile pāua, this was not observed. Survival over the 4 months was very high and was not affected by our experiment treatments. Although growth was not influenced by pH, it was clearly greater at 15 than 13 °C (73 vs. 44 μm d^−1^ SL), and physiological condition (CI_FWSW_) was greater at the lower temperature (Table 2). These growth rates are in the range of those predicted based on tag and recapture across a wide range of sites around New Zealand (Naylor, Andrew & Kim, 2006), and, along with the high survival rates, confirm the suitability of our experimental system for these investigations.

In contrast to our findings, Cunningham, Smith & Lamare (2016) found a negative influence of pH on survival and growth of juvenile pāua (5–13 and 30–40 mm SL) after 100 days (pH_NBS_ 8.1 > pH 7.6). One reason for these different findings may be the lower carbonate saturation states experienced by the pāua in the Cunningham, Smith & Lamare (2016) experiments: for example, at pH_NBS_ 7.6 and 16.5 °C, the Ω_Ar_ and Ω_Ca_ were 0.77 and 1.20, respectively. In our pH_T_ 7.66/15 °C treatment, Ω_Ar_ and Ω_Ca_ were considerably higher, at 1.07 and 1.66, respectively (Table 1). Additionally, the pāua were fed differently (Ramajo et al., 2016), and had different environmental histories (wild caught in Cunningham, Smith & Lamare (2016) vs hatchery spawned and reared from wild caught parents (*F*_1_) in this study). Reduced growth rates were noted for small (~15 mm SL) Pacific abalone (*H. discus hannai*) after 3 months at pH_NBS_ 7.9 and 7.7 (range of Ω_Ca_ = 1.27–1.77 and 1.90–2.63, respectively; Ω_Ar_ not reported; Li et al., 2018), and their CI_FWSW_ was reduced at pH_NBS_ 7.7 compared with pH_NBS_ 8.1 control animals (by 14.7%; Li et al., 2018). These differences illustrate the importance of considering seawater carbonate conditions when comparing between experiments, and the need for caution when generalising effects of one study (or species) to other situations.

### Shell morphology and integrity

Allometric relationships in pāua are quite consistent (see, Sainsbury, 1982), with SL strongly correlated to age, FW, and other measures. SWs were lighter and CI_SW:SL_ was significantly reduced at pH_T_ 7.66 compared with pH_T_ 8.00 (Fig. 2B; Table 2), with CI_SW:SL_ also lower at 13 °C than at 15 °C (Table 2). Cunningham, Smith & Lamare (2016) too found that pāua juveniles had lighter SWs for a given size in lower pH seawater. This pH effect may indicate that calcification is less effective in lower pH conditions, or that biomineralization has been disrupted (Fitzer et al., 2015; Lu et al., 2018). While we noted some differences in calcite content which might support the former (discussed below), our SEM investigations did not reveal any disruption to the shell ultrastructure.

Abalone shells have an inner nacreous aragonitic layer overlain by a prismatic layer, which is in turn overlain by a thin organic periostracum that protects the shell. The outer prismatic layer may be calcite (e.g. *H. kamtschatkana, H. rufescens*), aragonite (e.g. *H. asinina*, *H. glabra*), or a combination (*H. rubra*). In our *H. iris*, the prismatic layer is low-Magnesium calcite, and the nacre layer is aragonite (Table 3). Natural populations of adult *H. iris* exhibit variable calcification rates, shell layer thicknesses (Gray & Smith, 2004), and growth rates (Naylor, Andrew & Kim, 2006), which have been attributed to local differences in environmental conditions such as water temperature and wave exposure (McShane & Naylor, 1995; Naylor, Andrew & Kim, 2006). In our study, total shell thickness was not influenced by experimental treatment. We did, however, detect differences in thicknesses of the layers. At lower pH, the pāua had significantly thinner prismatic layers at two positions in the newly generated shell, and at one position in the pre-experiment shell (positions 3, 4 and 8; Figs. 3A and 3B). This layer was affected by temperature only at one position, where it was thinner at 15 °C than 13 °C (position 1; Figs. 3A and 3B). The nacre thickness in the newly deposited shell was significantly thicker at pH_T_ 7.66, and also at 15 °C, at one position only (position 3; Figs. 3A and 3B). Polar brachiopods also generated a thicker internal shell layer when grown under ocean acidification conditions (pH_NIST_ 7.54) for 7 months, demonstrating a plasticity in the calcification process that may help their response to future change (Cross, Harper & Peck, 2019). This nacreous layer can be repaired or augmented by the animal anywhere from the inside (see Cusack et al., 2013) and, because it is not (mostly) in direct contact with sea water, it is less vulnerable to mechanical breakage, chemical dissolution, or biological encrustation/erosion than the outer prismatic layer (although dissolution of the inner nacre layer has been noted for some species at reduced pH; Michaelidis et al., 2005; Melzner et al., 2011).

The prismatic layer’s outer surface is protected from direct contact with seawater by the overlying periostracum (Ries, 2011). However, abalone periostracum (thin among molluscs at about 0.2 μm thick) is often damaged in the high-energy environments they inhabit, and can also be affected by encrustation and biological borings of the shell. Visible erosion is often observed in wild adults (after 3 years in *H. fulgens*; Shepherd, Avalos-Borja & Ortiz Quinatilla, 1995). Greater surface etching overall (heavy and light) at lower pH was noted for the pre-experiment shell (Fig. 4B). The most striking effect was noted in the experiment shell, where heavy etching was minimal (~10%) in the ambient pH/13 °C treatment, but ranged from 60% to 80% in the remaining treatments, reflecting greater heavy etching at the lower pH and the higher temperature (Fig. 4A); the latter possibly reflecting the principle that dissolution rates are greater at higher temperature (Ries et al., 2016). Other studies have reported surface dissolution and thinning of the outer calcite layer with reduced pH. In small Pacific abalone incubated at pH_NBS_ 7.7 large areas of shell were without periostracum and extensive dissolution of the exposed calcite layer occurred (Li et al., 2018). Brachiopods (*Liothyrella uva* and *Calloria inconspicua)* also showed dissolution of their outer shell surface (Cross, Harper & Peck, 2019). Shells of *Mytilus edulis* were significantly thinner, had more injuries at the outer surface, and showed dissolution of the outer prismatic layer at pH 7.4 after 6 months (Ω_Ca_ = 1.15–1.21 and Ω_Ar_ 0.72–0.76; Bressan et al., 2014). In *M. edulis* shell properties have been shown to be altered under ocean acidification (increasing pCO_2_ from 380 to 1,000 μatm), through a reduced ability of the animal to control the ultrastructure (crystallographic orientation) of the calcite shell (Fitzer et al., 2014). These mussels produced calcite that was stiffer, harder and more brittle under ocean acidification conditions (Fitzer et al., 2015), which may potentially contribute to greater erosion.

In our low pH treatments, the average rate of thinning of the outer calcitic layer over the 4 month experiment was 0.1 mm. Since the layer is on average 0.4 mm thick, that rate of dissolution could remove the calcite altogether in about 16 months, exposing the aragonitic nacre to seawater. Farm-reared abalone exposed to high pCO_2_ levels can lose the prismatic layer altogether, even when small (Wright, 2011). In nature, where low pH seawater combines with abrasion of the periostracum and bioerosion, major shell thinning with consequent loss of strength and resilience to predation and wave action could become the norm for the adult life of these long-lived molluscs.

### Shell composition (mineralogy)

The shells of all the pāua in this experiment were composed of low magnesium calcite and aragonite (Table 3). The experimental shell contained 68–78% calcite (22–32% aragonite) on average, the pre-experiment shell 58–65% calcite (35–42% aragonite), and the spire shell 8–23% calcite (77–92% aragonite) (Table 3). This variable carbonate composition at different ages is to be expected (Auzoux-Bordenave et al., 2015). As both nacreous aragonite and prismatic calcite are stable minerals that retain their composition after deposition (at least in a time frame of months) we did not expect any difference in carbonate composition between individuals in the shell generated prior to the start of the experiment (Table 3). Indeed, only the shell which had grown during our experiment showed any treatment effect: 5–6% more wt% calcite was found at ambient pH than at pH_T_ 7.66, across both temperatures (Table 3). This likely reflects the significant erosion of the outer calcitic layer discussed above, possibly due to interaction with the other individuals crawling around in the chambers, and/or dissolution (although the seawater was oversaturated with respect to calcite in all treatments; Table 1). Whilst one might expect greater dissolution of aragonite at the saturation levels in our treatments (see Table 1), as discussed above, the aragonite nacre layer was not directly exposed to seawater. The wt% calcium in skeletal carbonate generally showed an opposite pattern to that of the wt% calcite (pH_T_ 7.66 > 8.00, 15 > 13 °C), in the experiment, pre-experiment and spire shells (albeit with some pH*temp interactions). Although we note that calcium can originate from both the aragonite and calcite shell layers, this pattern with pH seems counter to expectations of an effect on dissolution.

### Temperature and pH synergies

While significant temperature treatment effects varied with response parameter, higher growth, CI_SW:SL_, % calcium content and etching were noted at the slightly warmer temperature (15 > 13 °C). In particular, the influence of this relatively small difference in temperature on juvenile growth over only 4 months was striking (i.e. >2,000 μm more growth at 15 °C; Table 2). In several cases, a temperature effect was not apparent across both of the pH levels tested (i.e. when pH*temp < 0.05), with significant influences of temperature found only at ambient pH (e.g. % calcium, heavy etching of the experiment shell). Similarly, effects of pH were sometimes significant only at one temperature (e.g. only at 13 °C was % calcium content of the experimental and pre-experiment shell higher at pH_T_ 7.66 than ambient pH). This may in part be because of the differences in Ω_Ar_ in our lower pH treatments at the two temperatures—slight undersaturation at 13 °C and slightly above saturation at 15 °C (i.e. 0.992 and 1.066, respectively; Table 1). However, we do note that living organisms can be negatively affected at Ω_Ar_ > 1 (Waldbusser et al., 2015), and that calcification clearly occurred in all of our treatments, regardless of Ω_Ar_ (Ries, Cohen & McCorkle, 2009). The subtleties of effects of interactions between such small variations in temperature and ocean acidification (see Fitzer et al., 2014, 2015; Lu et al., 2018) and their relevance in a natural, more variable coastal environment remain to be determined. When temperature was elevated by 2 °C above ambient, disruption of crystallographic orientation by ocean acidification in *M. edulis* was greater (Fitzer et al., 2014), while effects on the shell stiffness, hardness and brittleness were reduced (Fitzer et al., 2015). Clearly, ocean acidification affects shell integrity, and the modifying influences of elevated temperature are complex and require more investigation. Indeed, Lu et al. (2018) showed that ocean acidification decreases the percentage of seawater DIC synthesised into shell carbonate in *M. edulis*, a response which is slightly greater at elevated temperature; they conclude that ongoing acidification and warming might interfere with calcification physiology through disrupting its ability to efficiently extract DIC from seawater.

## Conclusions

We have shown, by examining physiological, morphological and geochemical characteristics, that juvenile pāua can biomineralise both nacreous aragonite and prismatic calcite at End-Century low-pH projections. Mortality rates were negligible; only a small decrease in physical condition was observed, and the shell quality and composition did not change. However, post-depositional alteration of the shell, which cannot be controlled by the animal was noted in lowered pH conditions, with external dissolution reducing the thickness of the outer calcitic prismatic layer, particularly at the higher temperature. There is also evidence that the nacre layer has thickened at lowered pH and higher temperature.

Unlike most shellfish, the commercial value of pāua is in the quality of both the flesh and shells. The longer-term consequences of the effects noted here, especially worn and degraded shells, are particularly relevant. Consequences to resilience to physical stresses including predation and wave action, and how these may impact this important shell-fishery, remain to be investigated. While the effects we found on juveniles are small and mostly physical, when combined with widespread reports of effects on larval development for this genus (Parker et al., 2013) they are likely to provide a challenge in future conditions. A whole-life-cycle understanding of how pāua are influenced by ocean acidification and warming is needed to provide information on population-level consequences to this ecologically important species.

## Supplemental Information

10.7717/peerj.7670/supp-1 Supplemental Information 1 Raw data. This excel file contains worksheets of data on which the paper is based, to aid the review process. There are multiple worksheets, each with information on which part of the manuscript the data supports (e.g. cross referenced to figures, tables, or the results).

## Additional Information and Declarations

### Data Availability

The following information was supplied regarding data availability: The raw data are available as a Supplemental File.
